# Low invasive surfactant administration by videolaringoscopy: a feasibility study

**DOI:** 10.1186/s13052-025-02186-2

**Published:** 2025-12-22

**Authors:** Flavia Petrillo, Camilla Gizzi, Luigia Valenzano, Raffaele Manzari, Simona Marciante, Domenico Buongiorno, Vitoantonio Bevilacqua, Carlo Dani

**Affiliations:** 1 NICU “Di Venere” Hospital, Via Ospedale di Venere, 1, Bari, 70131 Italy; 2 Neonatology and NICU “Sant’Eugenio” Hospital - ASL RM2, Piazzale dell’Umanesimo 10, Rome, 00144 Italy; 3 Department of Electrical and Information Engineering (DEI), Polytechnic University of Bari, Via Edoardo Orabona, 4, Bari, 70126 Italy; 4 Apulian Bioengineering srl, Via delle Violette, 14, Modugno (BA), 70026 Italy; 5 Division of Neonatology, Careggi University Hospital of Florence, Largo Giovanni Alessandro Brambilla, Florence, 50134 Italy; 6 Department of Neurosciences, Psychology, Drug Research, and Child Health, University of Florence, Piazza San Marco, 4, Florence, 50121 Italy

**Keywords:** Videolaryngoscopy, Surfactant, LISA, RDS, Preterm infant

## Abstract

**Background:**

Less invasive surfactant administration (LISA) is a gentle emerging technique for surfactant administration through a thin catheter in infants receiving noninvasive ventilation. It seems to offer some advantages to very preterm infants. The purpose of the FRee of Invasiveness & Neonatal Delicate LISA (FRI&NDLI) study was to evaluate the feasibility and safety of using videolaryngoscopy for positioning the thin catheter tip beyond the vocal cords.

**Methods:**

We studied preterm infants with 24^+ 0^-32^+ 6^ weeks of gestation, born between September 2020 and January 2024. Data of infants with mild/moderate respiratory distress syndrome (RDS) who received surfactant through the FRI&NDLI procedure were recorded. Frequency of successes and incidence of related adverse effects of the procedure were also recorded.

**Results:**

Twenty-three infants with 28.9 ± 2.8 weeks of gestational age and a birth weight of 1255 ± 458 g who received 25 procedures were studied. No episodes of apnoea, bradycardia, or desaturation were recorded during the glottis visualization in videolaryngoscopy. Two episodes of bradycardia, 8 episodes of desaturation, and 1 episode of surfactant reflux were observed during surfactant administration.

**Conclusion:**

We found that the FRI&NDLI procedure was safe and associated with less adverse effects than previously reported for LISA procedure using direct laringoscopy. Our findings support the possibility of planning further studies to compare the effectiveness of FRI&NDLI and LISA procedure for surfactant administration in preterm infants.

## Introduction

Less Invasive Surfactant Administration (LISA), introduced in neonatology by Kribs et al. [1], is a technique that has gained prominence in recent years allowing the administration of surfactant using a thin catheter while the newborn breathes spontaneously on noninvasive ventilation. The thin catheter, due to its small diameter, allows the movement of the vocal cords during surfactant administration.

A recent meta-analysis showed that LISA reduces the need for mechanical ventilation (MV) and the incidence of death, bronchopulmonary dysplasia (BPD), and major prematurity complications compared to the classical INtubation-SURfactant-Extubation (INSURE) technique [2], described for the first time by Verder et al. in 1992 [3]. Studies from the German Neonatal Network seem to indicate that LISA expresses its maximum effectiveness when included in a bundle of procedures dedicated to the care of preterm infants, starting from the delivery room (DR) [4].

Despite being considered a gentle procedure, LISA requires the use of a laryngoscope and often a Magill forceps and remains an invasive maneuver, especially when it is performed without analgo-sedation. Moreover, it has been reported that LISA procedure can fail and have adverse effects, such as apnea, bradycardia, and surfactant reflux [5, 6].

A large retrospective analysis demonstrated that the first-attempt success rate of intubation is low (47.7%) and that the success rate is higher in infants weighing > 1.5 kg and in intubations performed by non-residents [7]. This is often due to the inability to quickly recognize the laryngeal structures or to poor visibility of the laryngeal space resulting from unskilled handling of the laryngoscope blade [6]. Obviously, these difficulties can influence the success of the intubation maneuver as well as that of the LISA procedure [8].

A recent study demonstrated that videolaryngoscopy can improve the glottic visualization with lower forces applied to upper airway tissues and reduced workload compared with direct laryngoscopy [9]. This finding is in agreement with a recent metanalysis which reported that videolaryngoscopy improves first attempt intubation success rates, with a number needed to treat (NNT) of 6 [10]. We then developed a procedure which uses videolaryngoscopy to visualize the glottis and which, together with other measures of care, was named the FRI&NDLI procedure (FRee of Invasiveness & Neonatal Delicate LISA).

Thus, the present feasibility study aimed to evaluate the hypothesis that it is operationally possible to safely use the FRI&NDLI procedure for administering surfactant in very preterm infants. This study is preliminary to the planning of a subsequent large randomized controlled trial to compare the efficacy and safety of the FRI&NDLI procedure with that of traditional LISA.

## Methods

All procedures were performed in compliance with relevant laws and institutional guidelines and in accordance with the Declaration of Helsinki. The study has been approved by the Ethics Committee “Policlinico Bari”, Bari, Italy. Preterm infants born at 24^+ 0^- 32^+ 6^ weeks of gestation at “Di Venere Hospital” (Bari, Italy) from September 1, 2020 to January 31, 2024 who received surfactant through the FRI&NDLI procedure were studied after parental informed consent. Entry criteria were the diagnosis of mild to moderate RDS requiring FiO_2_ ≥ 0.25 to maintain SpO_2_ ≥ 90% on noninvasive ventilation [Nasal Continuous Airway Pressure (NCPAP) or Nasal Intermittent Positive Pressure Ventilation (NIPPV)] or a Silverman score > 4 while on NCPAP (5–7 cmH_2_O) or NIPPV [Positive End-Expiratory Pressure (PEEP) 5–7 cmH_2_O; Peak Inspiratory Pressure (PIP) 15 cmH_2_O above PEEP; Respiratory Rate (RR) 40/min], good respiratory drive, and heart rate (HR) *≥* 120 bpm. Exclusion criteria were major congenital malformations, chromosomal disorders, inherited metabolic pathologies, and fetal hydrops. Diagnosis of RDS was based on the occurrence of tachypnoea, dyspnea, oxygen dependence, exclusion of other causes of respiratory failure, and the presence of a typical radiological pattern.

### The FRI&NDLI procedure

The following describes the step-by-step execution of the FRI&NDLI technique. *Step 1*: Infants were rotated by 180° inside the incubator, secured with wrapping, placed in a nest, and kept in anti-Trendelenburg position by inclining the incubator mattress of a 30° angle during and after the procedure, to prevent surfactant reflux. *Step 2*: the team, composed of two neonatologists and one nurse, prepared for the procedure. The nurse started the timer and marked the beginning of the procedure on the monitor. *Step 3*: the first neonatologist visualized the glottis using videolaryngoscopy (InfantView Acutronic™, Vyaire medical, Mettawa USA), inserted the thin catheter (LISAcath^®^ Chiesi Farmaceutici SpA, Parma, Italy or Surfcath^®^ Vygon, France) beyond the vocal cords, following the insertion depth criteria suggested by Dargaville et al. [11]. The videolaryngoscope blade was then quickly removed, holding the catheter firmly at the insertion depth previously marked by a sterile smear. Step 4. The second neonatologist connected the previously filled syringe with surfactant to the catheter cone and administered the surfactant (200 mg/kg of Curosurf^®^, Chiesi Farmaceutici SpA, Parma, Italy) prewarmed to room temperature in small boluses. The surfactant aliquots were ideally administered in synchrony with the newborn’s spontaneous breaths over 1–2 min. During surfactant administration the baby’s mouth was kept closed to ensure effective ventilation, and to prevent significant leaks and drop in pressure [12]. During the procedure, the nurse ensured the baby as much comfort as possible, using wrapping, nesting and holding. Step 5. The catheter was removed and the nurse marked the end of the procedure on the monitor. The baby was maintained in the anti-Trendelenburg position for 10 min to prevent surfactant reflux.

During and after the procedure, vital signs [systemic blood pressure (SBP), HR, and RR] were recorded using a multiparametric monitor (Infinity Delta Draeger, Lübeck, Germany).

A second dose of surfactant (Curosurf, 100 mg/kg) was allowed when Silverman score was > 4 or/and FiO_2_ > 30% [13] and was given using the FRI&NDLI technique when possible.

### Data collection

The following data were collected for each infant: gestational age, birth weight, sex, twinship, antenatal steroids, mode of delivery, Apgar score at 5 min, age at surfactant administration, need for mechanical ventilation, occurrence of BPD and intraventricular hemorrhage (IVH), and mortality. We recorded the duration of the FRI&NDLI procedure, and the incidence of apnoeas (> 10 s), desaturations (SpO_2_ < 85%), bradycardias (HR < 80 bpm), and surfactant reflux during the procedure. Main maternal pregnancies diseases, such as preterm premature rupture of membrane, placental abruption, and clinical chorioamnionitis, were also recorded.

### Statistical analysis

In the absence of previous studies to use as a reference, it was arbitrarily decided to study 25 procedures. The number of 25 procedures was determined based on logistical feasibility and resource availability during the study period, considering the limited number of eligible cases and the exploratory nature of this pilot phase, which aimed primarily to assess feasibility and safety rather than to perform a powered efficacy analysis. The clinical characteristics of infants were described by calculating their mean values and standard deviations, median and ranges or rates and percentages.

The primary endpoint was the percentage of cases in which surfactant was successfully administered with FRI&NDLI procedure. The secondary endpoint was the safety of the procedure which was evaluated measuring the occurrence of adverse effects (apnoeas, desaturations, bradycardias, surfactant reflux) during the procedure.

## Results

We analyzed 25 procedures which were performed in 23 infants with 28.9 ± 2.8 weeks of gestation and a birth weight of 1255 ± 458 g. Their clinical characteristics are reported in Table 1.

**Table 1:** Clinical characteristics of infants and main maternal pregnancy diseases

	*N* = 23
Gestational age (wks)	28.9 *±* 2.8
Birth weight (g) <10° percentile	1255 *±* 458 5 (21)
Female	10 (43)
Singleton	17 (74)
Antenatal steroids	21 (91)
Vaginal delivery	4 (17)
Apgar Score at 5 min	9 (5–10)
Noninvasive ventilation	23 (100)
Mechanical ventilation *≤* 72 h of age	4 (17)
Age at surfactant administration (h)	
First dose (n.23 infants)	7 (2–24)
Second dose (n.2 infants)	23 (22–24)
Bronchopulmonary dysplasia	1 (4)
Intraventricular haemorrhage	0
Sepsis	2 (8)
Death	2 (8)
**Main maternal pregnancy diseases**	
Preterm premature rupture of membranes	5 (21)
Placental abruption	5 (21)
Clinical chorioamnionitis	1 (4)

Data are expressed as Mean (± SD), number (%), or median (range)

The FRI&NDLI procedure had a 100% successful rate at the first attempt. No adverse effects were recorded during the glottis visualization in videolaryngoscopy. During surfactant administration 2 (8%) episodes of bradycardia, 8 (32%) of desaturation, and 1 (4%) of surfactant reflux were observed, while no episodes of apnea occurred (Table 2).

**Table 2:** Occurrence of adverse effects during the FRI&NDLI procedure

	*N*=25
Apnoea >10 s	0
Desaturation <85%	8 (32)
Bradycardia <80 bpm	2 (8)
Surfactant reflux	1 (4)
Mean duration of procedure (sec)	165 ±23

Data are expressed as number (%) or mean (±SD)

The mean duration of the procedure, from the insertion of the videolaryngoscope blade to the catheter extraction was 165 ± 23 s.

Two infants died due to the occurrence of a late onset sepsis.

## Discussion

The aim of this study was to evaluate the feasibility and safety of the FRI&NDLI procedure for administering surfactant in very preterm infants in order to plan a subsequent trial to compare this technique with traditional LISA.

We observed that FRI&NDLI procedure optimize surfactant administration since in our series all catheters were correctly placed on the first attempt. Using direct laryngoscopy for LISA procedure in 86 infants with gestational age < 32 weeks, de Kort et al. reported a 77% failure rate in catheter placement on the first attempt [14]. Specifically, in this study, a 5 Fr umbilical catheter was used for surfactant administration in the first treated infants, then the LISAcath (Chiesi Farmaceutici, Parma, Italy) was adopted which simplified the procedure. Moreover, the failure rate was reduced to 33% when the procedure was performed by experienced neonatologists [14]. Several studies in the literature emphasize that LISA must be performed by experienced neonatologists to ensure its success on the first attempt [14–16]. Indeed, more attempts cause significant stress for the infant, especially if not adequately sedated. Unlike our study, Ives and colleagues using videolaryngoscopy for LISA procedure, reported a success rate on the first attempt of only 47%, being the most common reason for an unsuccessful first attempt an inability to advance the tubes despite a view of the cords due to the large handle/blade of the videolaringoscopy, poor hand–eye coordination, and difficult angle of insertion [16]. In our pilot study, all neonatologists who performed the procedure had at least five years of post-specialization experience in the NICU. In our setting, while operator expertise and center-specific factors may have contributed to the 100% first-attempt success rate, we believe that the favourable performance of the FRI&NDLI procedure was largely attributable to the enhanced glottic visualization provided by our extremely versatile video laryngoscope, which allowed easier and more accurate catheter placement than traditional direct laryngoscopy.

Another relevant aspect that made the procedure gentle and well tolerated was that videolaryngoscopy improved visualization of the glottis with less force applied to the upper airway tissues and a reduced workload compared with direct laryngoscopy [9].

Indeed, we observed no bradycardias, apneas, or desaturations during glottic visualization, whereas a variable rate of these side effects with respect to the glottic visualization procedure is reported in the literature, often related to the level of experience of the neonatologists performing the technique [9].

During the FRI&NDLI procedure, using the correct precautions which include non-pharmacological sedation, we observed a lower complication rate than reported in the literature for the LISA approach using direct laryngoscopy [5, 6, 17]. There are very few clinical studies detailing side effects related to surfactant administration during LISA. Hertig, referring to the experience of the German network, emphasized once more that the technique should be performed by experienced neonatologists to minimize side effects [16]. He also reported that failure to insert the catheter beyond the vocal cords on the first attempt, significant surfactant reflux, acute desaturations, bradycardia, and/or the need for positive pressure ventilation during LISA were observed in less than 10% to more than 30% of LISA/MIST manipulations [18]. Lista et al. studied 18 infants with a mean gestational age of 30 *±* weeks and found that the occurrence of bradycardia and desaturations during the procedure was 43% and 52%, respectively [15].

In addition, studies with continuous monitoring of cerebral regional saturation by near-infrared spectroscopy (NIRS) suggested that laryngoscopy is more often a source of side effects than surfactant instillation itself [19]. In our experience, the use of videolaryngoscopy during LISA appeared to be associated with fewer procedure-related side effects compared with traditional direct laryngoscopy, likely due to improved visualization of the glottis. However, given the heterogeneity among published studies and procedural conditions, this observation should be interpreted with caution and confirmed in larger comparative trials.

It is interesting to underline that our patients did not need sedo-analgesia as the manoeuvre was quick and well tolerated, and none of patients experienced apnoea during the procedure. This, in agreement with previous studies, is probably due to the use of videolaryngoscopy which facilitates catheter placement [20, 21]. Therefore, FRI&NDLI procedure could contribute to overcome the issue of sedoanalgesia during LISA procedure.

## Conclusions

In conclusion, this feasibility study demonstrated that the FRI&NDLI technique allowed to perform the LISA procedure with a 100% success rate, high tolerability and limited incidence of adverse effects, despite the absence of sedoanalgesia. Although our pilot study yielded these encouraging results, our findings may not be directly generalizable to units with different levels of experience or training. Further multicenter investigations are warranted to confirm their reproducibility across diverse clinical setting and support the planning of a randomized controlled trial to compare the efficacy and safety of the FRI&NDLI procedure with that of traditional LISA.

## Data Availability

The data that support the findings of this study are available from the corresponding author upon reasonable request.

## Abbreviations

LISA: Less Invasive Surfactant Administration
FRI&NDLI: Free of Invasiveness & Neonatal Delicate LISA
RDS: Respiratory Distress Syndrome
MV: Mechanical Ventilation
BPD: Bronchoplumonary Dysplasia
INSURE: INtubation-SURfactant-Extubation
DR: Delivery Room
NNT: Number Needed to Treat
NCPAP: Nasal Continuous Airway Pressure
NIPPV: Nasal Intermittent Positive Pressure Ventilation
NIRS: Near-Infrared Spectroscopy
PEEP: Positive End Expiratory Pressure
PIP: Peak Inspiratory Pressure
SBP: Systemic Blood Pressure
HR: Heart rate
RR: Respiratory rate
IVH: Intraventricular hemorrhage
